# Comparison of clinical characteristics of a pediatric cohort with combined pituitary hormone deficiency caused by mutation of the *PROP1* gene or of other origins

**DOI:** 10.1007/s42000-023-00510-1

**Published:** 2023-12-26

**Authors:** Agata Zygmunt-Górska, Małgorzata Wójcik, Aleksandra Gilis-Januszewska, Anna Starmach, Mirosław Bik-Multanowski, Jerzy B. Starzyk

**Affiliations:** 1 Department of Pediatric and Adolescent Endocrinology, University Children’s Hospital in Cracow, Cracow, Poland; 2 Department of Pediatric and Adolescent Endocrinology, Chair of Pediatrics, Pediatric Institute, Jagiellonian University Medical College, Ul. Wielicka 265, 30-663 Cracow, Poland; 3 Chair and Department of Endocrinology, Jagiellonian University Medical College, Cracow, Poland; 4 Department of Medical Genetics, Jagiellonian University Medical College, Cracow, Poland

**Keywords:** Pituitary, Combined pituitary hormone deficiency, *PROP1*

## Abstract

**Abstract:**

The most commonly identified genetic cause of combined pituitary hormone deficiency (CPHD) is *PROP1* gene mutations. The aim of the study was to compare selected clinical features of patients with CPHD caused by variants of the *PROP1* gene (CPHD-PROP1) and patients with inborn CPHD of other etiology (CPHD-nonPROP1).

**Material and methods:**

The retrospective analysis included childhood medical records of 74 patients (32 female) with CPHD, including 43 patients (23 female) with the mutation in the *PROP1* gene.

**Results:**

Patients with CPHD-PROP1 compared to the CPHD-nonPROP1 presented with the following: significantly higher median birth weight (0.21 vs. − 0.29 SDS, *p* = 0.019), lower growth velocity within 3 years preceding growth hormone administration (− 2.7 vs. − 0.8 SDS, *p* < 0.001), higher mean maximal blood concentration of growth hormone within the stimulation process (1.2 vs. 1.08 ng/mL, *p* = 0.003), lower TSH (1.8 vs. 2.4 µIU/mL, *p* < 0.001), significantly lower prolactin concentrations (128 vs. 416.3 µIU/mL, *p* < 0.001), and less frequent typical signs of hypogonadism at birth in boys (*n* = 6; 30% vs. *n* = 12, 54%, *p* < 0.001). Secondary adrenal insufficiency was less frequent in CPHD-PROP1 (20 vs. 25 cases, *p* = 0.006) and occurred at a later age (13.4 vs. 10.4 years). MRI of the pituitary gland in CPHD-PROP1 revealed a small pituitary gland (21 cases), pituitary gland enlargement (eight cases), and one pituitary stalk interruption and posterior lobe ectopy, while it was normal in nine cases.

**Conclusion:**

Patients with the *PROP1* mutations present a clinical picture significantly different from that of other forms of congenital hypopituitarism. Certain specific clinical results may lead to the successful identification of children requiring diagnostics for the *PROP1* gene mutation.

## Introduction

Combined pituitary hormone deficiency (CPHD) is characterized by a deficiency of at least two of six anterior pituitary hormones, namely, growth hormone (GH), thyroid-stimulating hormone (TSH), prolactin (PRL), adrenocorticotropic hormone (ACTH), and at least one gonadotropin (LH, FSH). The majority of cases of CPHD in pediatric populations are inborn. They are the results of impaired brain development due to congenital brain malformation or other genetic conditions. CPHD may also occur due to damage to the region of the hypothalamic-pituitary system resulting from, inter alia, trauma, tumor development, and irradiation, although this is more frequent in older age groups [1, 2]. Genetically determined familial and sporadic forms of CPHD may be caused by an abnormal structure of genes encoding transcription factors such as *PROP1*, *POU1F1*, *HESX1*, *LHX3*, and *LHX4* which influence the formation of the pituitary gland by acting according to a specific temporal and spatial pattern [3]. Pathogenic gene variants (mutations) of *PROP1* are thought to be the most common cause of genetically determined CPHD (up to 50% of cases) [4, 5]. The *PROP1* gene is located on the long arm of the fifth chromosome pair (5q35) [4]. The most common mutation is a deletion of two base pairs described as c.301_302del. The deletion results in a frame-shift mutation and premature termination of transcription. The resulting protein is deprived of the ability to bind to DNA, and, consequently, the activation of transcription is stopped [6, 7]. Mutations inactivating *PROP1* are manifested by both abnormal pituitary morphology and loss of its function [6, 7], causing GH, TSH, PRL, LH, and FSH deficiency. The studies conducted so far show that the mutation in the *PROP1* gene does not affect the development of pituitary corticotrope cells [6–9]. Depending on the cause of the disorder and the age of the patient, the symptoms of CPHD may have different clinical presentations, indications, and symptoms. In the neonatal period and in early infancy, the most characteristic signs include hypoglycemia, prolonged jaundice, and features of hypogonadism in boys (cryptorchidism, reduced testicle volume, and penile underdevelopment) [8, 9]. At preschool or school age, a decrease of growth velocity and short stature dominates. Single cases of congenital CPHD diagnosed in adolescents manifested as an absence of puberty [8, 10, 11]. The abovementioned *PROP1* mutation seems to be the most common cause of CPHD in Central and Eastern Europe, and it generally defines a specific sequence of clinical symptoms as well as specific changes in MRI imaging of the pituitary gland [11–14]. Patients with CPHD caused by the c.301_302del mutation require long-term surveillance of adrenal function, as they may develop symptoms of adrenal insufficiency in the second or third decade of life [10]. While knowledge concerning the evolution of the radiological imaging of CPHD continues to improve, the data are incomplete on patients’ clinical characteristics [6, 11, 15]. Even less is known about the genotype–phenotype correlation in CPHD with a non-*PROP1* mutation etiology [10, 15].

The initial aim of the study was to compare selected clinical features of patients with CPHD caused by variants of the *PROP1* gene (CPHD-PROP1) and patients with inborn CPHD of other etiology (CPHD-nonPROP1). The study’s second aim was to verify the hypothesis that on the basis of clinical characteristics, patients could be selected for genetic diagnostics of the PROP1 gene mutation.

## Material and methods

The retrospective analysis included childhood medical records of 74 patients (42 male and 32 female) with CPHD. Additionally, those parents of the patients living and who were willing to participate in the CPHD-PROP1 study were included in the second part, namely, genetic examination. The study analyzed clinical parameters such as age at time of diagnosis, first signs and symptoms, the order of appearance of pituitary disorders, their severity, and anthropometric parameters. This was achieved by carrying out a comparison between the groups, CPHD-PROP1 and CPHD-non-PROP1.

Genetic analysis of the *PROP1* gene was performed using PCR amplification of all three exons of the *PROP1* gene; subsequent screening analysis of the obtained amplicons was performed by means of DHPLC chromatography (Wave Transgenomic, USA) to detect presumed structural DNA variants. The direct sequencing was used to identify the detected variant.

Growth was measured with a Harpenden stadiometer (accurate to within 1 mm). Body weight was measured with a medical scale with an accuracy of 0.1 kg. The available anthropometric data are presented in the results section as standardized values (standard deviation score, SDS). The data were interpreted in relation to the national norms of height, weight, and BMI [16]. Growth velocity (GV) was calculated on the basis of two height measurements over a minimum period of 3 months. All measurements were performed before the introduction of human recombinant GH treatment. Puberty was assessed according to the Marshall and Tanner scale [17, 18]. Testicular size in boys was assessed according to the Prader orchidometer scale. Hormone concentrations were determined in collected serum samples using radiometric and immunometric methods. Growth hormone deficiency was confirmed if the highest GH concentration was < 10 ng/mL in two of the stimulation tests (L-dopa 300 mg/m2 and propranolol 1 mg/kg, insulin 0.015–0.03 IU/kg, glucagon 0.03–0.05 mg/kg, or clonidine 75–150 µg/m2). Central hypothyroidism was diagnosed based on a decreased concentration of thyroxine (total or free) in the absence of a compensatory increase in TSH concentration. In patients with suspicion of central hypothyroidism, the diagnosis was confirmed based on the result of the test with recombinant TRH (5–7 µg/kg). Secondary adrenal insufficiency was diagnosed based on the measurement of cortisol concentrations before and after the administration of insulin or glucagon (in doses as in the GH stimulation tests). Secondary adrenal insufficiency was diagnosed when morning cortisol concentration was below 50 ng/mL and ACTH concentration below 40 pg/mL. In the case of inconclusive results, some patients underwent a short test with low dosage of synthetic ACTH (cosyntropin 1 µg). Secondary adrenal insufficiency was confirmed if blood cortisol concentration after stimulation did not exceed 180 ng/mL [19]. Secondary hypogonadism was suspected in the absence of breast enlargement in girls and testicular enlargement in boys up to the age of 13 and 14 years of age, respectively. In these cases, LH, FSH, estradiol, and testosterone concentrations were measured before and after administration of a short-acting gonadoliberin analog (GnRH, 100 µg) [7] (for details see Table 3). Magnetic resonance imaging of the hypothalamic-pituitary region was performed using 1.5 T scanners with 3 mm layers in the sagittal and coronary planes, SE T1, and T2 before and after contrast administration. In 18 patients, only computed tomography of the pituitary gland was performed during pediatric follow-up [20]. They were diagnosed before MRI was implemented in routine practice, and it was not possible to perform MRI later.

The study protocol was approved by the Bioethics Committee of the Jagiellonian University (no. KBET/100/B/2010 of June 25, 2010) and was performed in accordance with the principles contained in the Declaration of Helsinki.

### Statistical analysis

The statistical software STATISTICA 10.0 PL was used for the statistical analysis of results. Data were described using arithmetic mean and standard deviation. In order to compare the two data sets, the Student’s *t*-test for unrelated samples was used, and in the absence of a normal distribution of the data, the Mann–Whitney *U* test was implemented. To compare categorical values χ^2^ test was used. A two-tailed *p*-value < 0.05 was considered statistically significant.

## Results

**Fig. 1:**
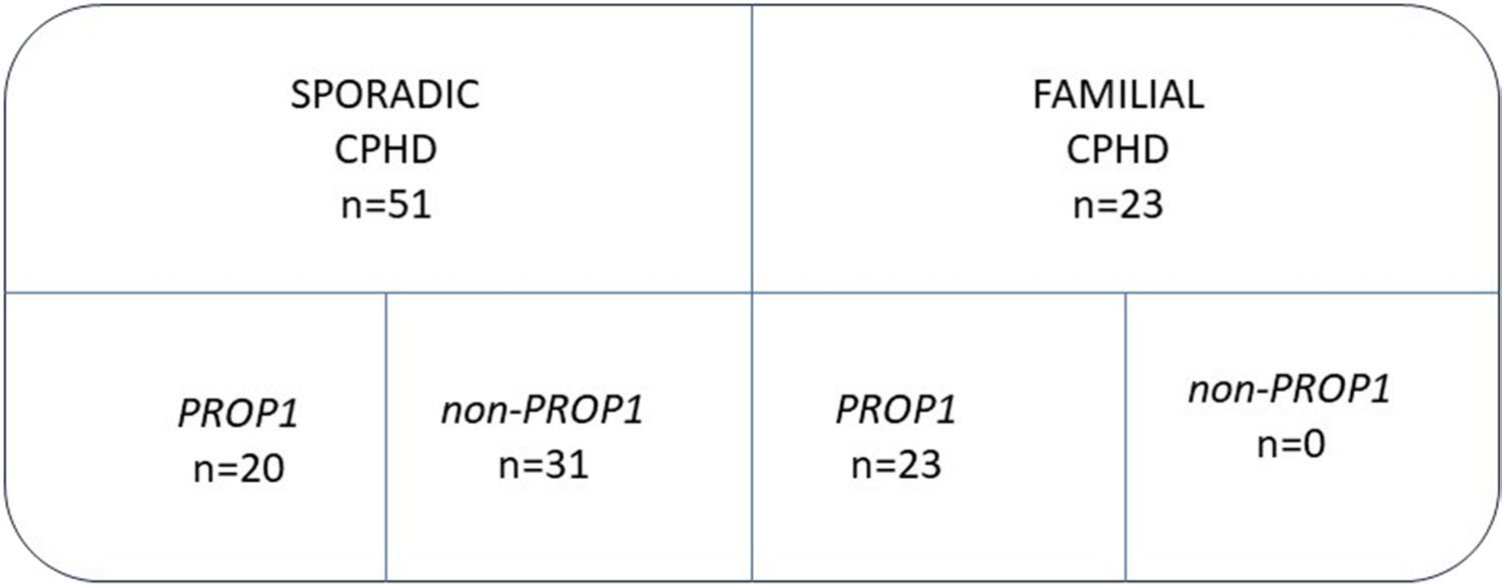
Structure of the study group

In 51 patients, CPHD occurred sporadically, and in 23 (31%), CPHD revealed familial occurrence with two affected siblings in seven families and three affected siblings in three families. In 43 patients (23 females and 20 males), mutations in the *PROP1* gene were identified (CPHD-PROP1 group), whereas in 31 (nine females and 22 males) no *PROP1* mutation was detected (CPHD-nonPROP1 group) (Fig. 1). In the group of patients with mutations of the *PROP1* gene, gender distribution was equal, but in the group without mutations, male sex was predominant (46.5 vs. 71% *p* = 0.001). There was a significant difference in mean age at diagnosis of CPHD in both groups, namely, 5 years in CPHD-PROP1 and 8 years in CPHD-nonPROP1 patients (*p* = 0.006).

### The PROP1 mutations

The most frequent mutation was the deletion c.301_302del (p.Leu102fs), which was homozygous in 23 patients (genotype c.301_302del/c.301_302del). The second biggest group of patients (14 children) revealed complex heterozygosity with mutations c.301_302del and c.150_151del (p.Gly52fs). In four patients, a homozygous mutation, c.150_151del/c.150_151del, was confirmed. In addition, two less common mutations, c.295C > T (p.Arg99Ter) and c.349 T > A (p.Phe117Ile), were detected in two complex heterozygotes carrying also the c.301_302del mutation. In 25 parents (13 mothers and 12 fathers) of CPHD-PROP1 children, a single mutant *PROP1* allele was found. In 10 cases, both parents were carriers, and in 13 cases, the presence of the mutation was confirmed in only one parent (Table 1).

**Table 1:** Characteristics of CPHD-PROP1 families regarding the type of mutation

Family no	Number of family members participating in the study: (sex)	*PROP1* mutation (patient)	Carrier: single-allele mutation in parents
1	2: P (♀, ♂)	c.301_302del/c.1 50_151del	
2	2: P (♀, ♀)	c.301_302del/c.301_302del	
3	2: P (♀, ♂)	c.301_302del/c.150_151del	
4	3: P (♀, ♀,♂)	c.301_302del/c.301_302del	
5	2: P (♀, ♂)	c.301_302del/c.301_302del	
6	4: P (♂, ♂)	c.301_302del/c.301_302del	M-c.301_302del
	M, F		F–c.301_302del
7	5: P (♀, ♀, ♂)	c.301_302del/c.301_302del	M-c.301_302del
	M, F		F–c.301_302del
8	4: P (♀, ♂)	c.301_302del/c.150_151del	M-c.150_151del
	M, F		F–c.301_302del
9	5: P (♂, ♂, ♂)	c.301_302del/c.301_302del	M-c.301_302del
	M, F		F–c.301_302del
10	3: P (♀)	c.301_302del/c.349 T > A	M-c.301_302del
	M, F		F–c.349 T > A
11	3: P (♀)	c.150_151del/c.150_151del	M-c.150_151del
	M, F		F–c.150_151del
12	3: P (♀)	c.301_302del/c.150_151del	M-c.150_151del
	M, F		F–c.301_302del
13	3: P (♂)	c.301_302del/c.301_302del	M-c.301_302del
	M, F		F–c.301_302del
14	3: P (♂)	c.301_302del/c.295C > T	M-c.301_302del
	M, F		F–c.295C > T
15	3: P (♂)	c.301_302del/c.150_151del	M-c.150_151del
	M, F		F–c.301_302del
16	2: P (♀)	c.301_302del/c.150_151del	F–c.150_151del
	F		
17	2: P (♀)	c.301_302del/c.301_302del	M-c.301_302del
	M		
18	3: P (♀, ♂)	c.301_302del/c.301_302del	M-c.301_302del
	M		

*P* patient CPHD-PROP1, *M* mother, *F* father

The clinical characteristics of CPHD-PROP1 and CPHD-nonPROP1 are presented in Table 2.

**Table 2:** Most important differentiating signs and symptoms in patients with CPHD-PROP1 and CPHD-nonPROP1

	CPHD-PROP1 (*N* = 43)	CPHD-nonPROP1 (*N* = 31)	
Sex	♀ 23 (53%), ♂ 20 (47%)	♀ 9 (29%), ♂ 22 (71%)	*p* = 0.001#
Median birth weight SDS	0.21 (− 0.1; 0.52)	− 0.29 (− 0.7; 0.7)	*p* = 0.019*
Cases of birth weight > 2 SD	2 (5%)	0	
Cases of birth weight < − 2 SD	0	2 (6%)	
Complications in the course of pregnancy and delivery			
Abnormal position of the fetus	0	14 (45%)	
Maternal gestosis	0	1 (3%)	
Premature delivery	1 (2%)	3 (10%)	
Delivery type			
Vaginal	39 (91%)	27 (87%)	
Cesarean section	4 (9%)	4 (13%)	
First symptom of MPHD			
Hypoglycemia in the neonatal period	0	5 (16%)	
Short stature	43 (100%)	26 (84%)	
Mean height at the time of diagnosis in short stature patients	− 4.1 SDS	− 4.3 SDS	*p* = 0.64**
Average growth velocity within 3 years preceding growth hormone therapy	− 2.7 SDS	− 0.8 SDS	
Median age at diagnosis of secondary hypothyroidism (years)	5.0 (0.1; 8)	8.3 (3; 12)	*p* = 0.006*
Symptoms of hypogonadism at birth (non-descended testes and/or micropenis)	*n* = 6 (30%)	*n* = 12 (54%)	*p* < 0.001#
Median age at diagnosis of GH deficiency (years)	5 (0.1; 7)	8 (3.5–14)	*p* = 0.006*
Median age at start of GH treatment (years)	6 (0.3; 12)	9 (2.2–13)	*p* < 0.001*
Mean age at diagnosis of ACTH deficiency (years)	13.4 ± 2.4	10.4 ± 2.9	*p* = 0.2**

Continuous variables are presented as mean ± SD or median (min:max)

^#^*χ*^2^ test

^*^Mann–Whitney *U* test

^**^Student’s *t*-test

### Characteristics of pregnancy and the neonatal period

Pregnancy and delivery were uncomplicated in all patients with CPHD-PROP1, with the exception of one premature delivery in the 36th week of gestation. In contrast, in the CPHD-nonPROP1 group, there were 14 cases of abnormal position of the fetus (buttock, foot, or transverse) (Table 3). In two cases, the disorder was related to twin delivery. Additionally, there was one case of maternal gestosis and three cases of premature births, including one in the 30th week of pregnancy. Most of the deliveries were via the birth canal and in eight cases by cesarean section (four in each of the two groups). Despite the birth weight of 95% of the children being normal, ranging from − 2.0 to 2.0 SDS, patients with CPHD-PROP1 had a significantly higher median birth weight compared to CPHD-nonPROP1 (0.21 vs. − 0.29 SDS, *p* = 0.019). Two newborns with a birth weight above 2 SDS were found in the CPHD-PROP1 group, and two with a birth weight less than − 2 SDS were identified in the CPHD-nonPROP1 group. Hypoglycemia was diagnosed in five CPHD-nonPROP1 patients in the neonatal period: this resulted in further examinations, which led to the diagnosis of hormonal deficits. Additionally, retrospective analysis of the medical records of patients who were diagnosed later in life due to short stature revealed that in a further seven patients (three cases in the CPHD-PROP1 and four cases in the CPHD-nonPROP1 group), transient hypoglycemia was also present in the neonatal period, but no further diagnostic steps were taken during this period.

**Table 3:** Characteristics of hormonal pituitary function at the moment/time of diagnosis in patients with CPHD with and without *PROP1* gene mutation

	CPHD-PROP1 (*N* = 43)	CPHD-nonPROP1 (*N* = 31)	
Maximal blood GH concentration^$^ (ng/mL)	1.2 ± 0.8	1.08 ± 0.5	*p* = 0.003**
Mean blood IGF-1 concentration (ng/mL)	40.2 ± 12.0	42.6 ± 13.9	*p* = 0.1**
Mean blood TSH concentration (µIU/mL)	1.8 ± 0.8	2.4 ± 1.6	*p* < 0.001**
Mean blood fT4 concentration (µIU/mL)	8 ± 2.3	7 ± 2.3	*p* = 0.9**
Mean blood TSH concentration in the TRH stimulation test (µIU/mL)	7.6 ± 14.7	3.96 ± 11.4	*p* = 0.006**
Mean blood LH concentration (µIU/mL)	0.4 ± 0.36	0.8 ± 0.3	*p* = 0.6**
Mean blood FSH concentration (µIU/mL)	0.5 ± 0.5	1.5 ± 0.8	*p* = 0.036**
Mean blood LH concentration in the GnRH stimulation test (µIU/mL)	0.6 ± 0.7	3.9 ± 1.0	*p* = 0.25**
Mean blood FSH concentration in the GnRH stimulation test (µIU/mL)	0.7 ± 0.3	3.8 ± 4.0	*p* = 0.014**
Median blood PRL concentration (µIU/mL)	130.8 ± 129	416.3 ± 493	*p* = 0.0010*

Continuous variables are presented as mean ± SD or median (min, max)

^$^In the stimulation tests

^*^Mann–Whitney *U* test

^**^Student’s *t*-test

### Growth

In all CPHD-PROP1 patients, differential diagnosis was initiated due to short stature. In five patients of the CPHD-nonPROP1 group, diagnosis was made due to hypoglycemia in the neonatal period (before growth failure occurred). With the exception of these five cases, diagnostic procedure was also started due to short stature. No statistically significant difference was observed between CPHD-PROP1 and CPHD-nonPROP1 patients in regard to their mean height at the moment of diagnosis (− 4.1 SDS and − 4.3 SDS, respectively, *p* = 0.64). The average growth velocity within 3 years preceding growth hormone therapy was lower in the CPHD-PROP1 patients than in the CPHD-nonPROP1 group (− 2.7 vs. − 0.8 SDS, *p* < 0.001).

### Growth hormone

The diagnosis of GH deficiency was made for both CPHD-nonPROP1 and CPHD-PROP1. The mean maximal blood concentration of GH in the stimulation tests in the CPHD-PROP1 group was higher than in the CPHD-nonPROP1 group (mean 1.2 ± 0.8 vs. 1.08 ± 0.5 ng/mL, *p* = 0.003).

### Thyroid axis

All patients in both groups were diagnosed with secondary hypothyroidism. The two groups did not differ in terms of total and free thyroxine concentrations (CPHD-PROP1 and CPHD-nonPROP1 patients: mean 5.5 ± 1.6 vs. 4.7 ± 1.9 µg/dL; *p* = 0.47 and mean 8 ± 2.3 vs. 7 ± 2.3 pmol/L; *p* = 0.9, respectively). However, basic TSH concentrations were significantly lower in the CPHD-PROP1 group (mean 1.8 ± 0.8 vs. 2.4 ± 1.6 µIU/mL, *p* < 0.001), as well as the mean maximal TSH concentration in the TRH test (mean 7.6 ± 14.7 vs. 3.96 ± 11.4 µIU/mL, *p* = 0.006). The mean age at diagnosis of secondary hypothyroidism in CPHD-PROP1 patients was significantly lower compared to the CPHD-nonPROP1 group (5.2 vs. 8.0 years, *p* = 0.006).

### Gonadal axis

Symptoms typical of hypogonadism at birth (non-descended testes and/or micropenis) were less frequent in CPHD-PROP1 boys (*n* = 6/30% vs. *n* = 12/54%; *p* = 0.0002). Spontaneous maturation did not occur in any patient. The GnRH test confirmed hypogonadotrophic hypogonadism. At the age typical for puberty, testosterone and estradiol concentrations in both groups were low and corresponded to the values of these hormones in prepubescent patients. The mean basic gonadotropin concentrations in CPHD-PROP 1 and CPHD-nonPROP1 patients were low (mean LH 0.4 ± 0.36 vs. 0.8 ± 0.3 µIU/mL, *p* = 0.6) and FSH (mean 0.5 ± 0.5 vs. 1.5 ± 0.8 µIU/m L, *p* = 0.036). The mean LH and FSH concentrations after GnRH stimulation were lower in CPHD-PROP1 patients (mean LH 0.6 ± 0.7 vs. 3.9 ± 1.0 µIU/mL, *p* = 0.25; mean FSH 0.7 ± 0.3 vs. 2.7 ± 4.0 µIU/mL, *p* = 0.014).

### Prolactin

Concentrations of PRL were significantly lower in CPHD-PROP1 patients (mean 128 ± SD 129 vs. 416.3 ± 439 µIU/mL, *p* < 0.001). In the CPHD-PROP1 group, prolactin concentration was decreased in 18 of 20 patients (data are not available for the rest of the group). In the CPHD-nonPROP1 group, a decreased prolactin concentration was confirmed in seven of 12 patients (data are not available for the rest of the group due to the retrospective character of the study). The mean age of the diagnosis of prolactin deficiency was lower in the CPHD-PROP1 group compared to the CPHD-nonPROP1 (5.0 vs. 8.0 years, *p* = 0.006).

### Adrenal axis

Secondary adrenal insufficiency was diagnosed in all five CPHD-nonPROP1 patients who presented with overt hypoglycemia in the neonatal period. In other patients at the time of diagnosis, the adrenal axis was assessed and in those who presented with normal morning cortisol and/or cortisol after stimulation with synthetic ACTH (cosynthropin 1 µg i.v.) diagnostic procedures were repeated cyclically. Finally, secondary adrenal insufficiency was diagnosed in 20 CPHD-PROP1 and in 25 CPHD-nonPROP1 patients (*p* = 0.006). Secondary adrenal insufficiency in the CPHD-PROP1 group occurred at a later age (13.4 vs. 10.4 years, respectively).

### Pituitary imaging

In the CPHD-PROP1 group, in those patients who underwent MRI of the pituitary gland, the examination at the time of diagnosis revealed a small pituitary gland in 21 cases (including one case of pituitary stalk interruption and posterior lobe ectopy) and pituitary gland enlargement in eight cases. In nine cases, the result of the examination was normal.

In the CPHD-nonPROP1 group, MRI revealed anterior lobe hypoplasia in 16 patients, pituitary stalk interruption and ectopy of the posterior lobe in 10 patients, anterior lobe hypoplasia and pituitary stalk interruption without posterior lobe ectopy in five patients, enlargement and heterogeneous signal of the pituitary gland in one case, and normal pituitary gland in two patients. In 15 patients of the CPHD-PROP1 group, MRI was repeated after 10 years. In 11 cases, the size of the pituitary gland had decreased, in one case it remained unchanged, and in three cases it increased. The CT scan was the only imaging examination of the pituitary gland in 18 patients, including four CPHD-PROP1 individuals. It suggested pituitary hypoplasia in 14 CPHD-nonPROP1 and in two CPHD-PROP1 patients. Moreover, pituitary microadenoma (4 mm in diameter) and pituitary nodular enlargement with the presence of microcysts and calcifications were described in two CPHD-PROP1 patients aged 8.8 and 18.4 years, respectively.

### Discussion

The estimated global prevalence of CPHD is 1 in 8000 (https://www.ghr.nlm.nih.gov). It may be a disorder of known genetic or of idiopathic etiology. To date, 30 genes have been reported to be involved in the pathogenesis of CPHD [10]. In this study, *PROP1* gene variants were found in 43/74 patients (58%) and were, as previously stated by other authors, the most frequent genetic etiology of CPHD [11]. A much higher percentage was found in a Lithuanian cohort (70%) [21]. In comparison to this, in Japan, non-*PROP1* mutation was not confirmed in any of the studied patients [22]. *PROP1* mutation prevalence is higher in familial rather than in sporadic cases, as was also found in the current study (23/23 100% in familial vs. 20/51 39% in sporadic) [23–26]. In the study group, we observed significant male predominance in the CPHD-nonPROP1 group. This is consistent with observations made by other authors describing similar cohorts [27]. The origin of this phenomenon remains unclear; however, it has been hypothesized to be due to a role of X-linked recessive genes, sex chromosome-environmental interaction, or an unexplained male susceptibility to perinatal insult [28]. The detailed assessment of the study group in the present study revealed some significant differences between CPHD-PROP1 and CPHD-nonPROP1 patients. The birth weight of most children with CPHD was normal, although patients with CPHD-PROP1 had a higher mean birth weight compared to the CPHD-nonPROP1 group. This is consistent with the observation of other authors [26, 28, 29]. In the present study, among patients with CPHD-PROP1, no cases of abnormal positioning of the fetus at delivery were found. On the contrary, in the CPHD-nonPROP1 group, there were as many as 14 of 37 patients (38%) with abnormal position of the fetus at delivery. The latter was also demonstrated by Diwaker et al., who found that breech presentation was higher in PSIS-CPHD than in *POU1F1/PROP1*-CPHD (44.4 vs. 5.5%) and concluded that breech presentation in PSIS is likely due to pituitary stalk interruption rather than to hormonal deficiency [27]. This confirms the hypothesis that in the early stage of intrauterine development, factors other than the *PROP1* mutations, which result in an alteration of the development of the central nervous system, including the pituitary gland, have a stronger impact on fetal growth and the course of labor [30]. As was previously demonstrated in CPHD-PROP1, TSH and GH deficiencies have a tendency to occur in early childhood, whereas gonadotropin and corticotropin deficiencies manifest later in life [11, 31]. In five patients of the CPHD-nonPROP1 group, diagnosis was made due to hypoglycemia in the neonatal period (before growth failure had occurred) probably because of concomitant ACTH deficiency. In CPHD-PROP1 mutation patients, since ACTH deficiency occurs variably as the patient grows older, symptoms of adrenal failure did not occur in the neonatal period. Another interesting finding may be the fact that severe GH deficiency was found in both groups (in the CPHD-PROP1 group compared to the CPHD-nonPROP1 1.2 vs. 1.08 ng/mL, *p* = 0.003). The reason for this phenomenon is unknown, although the mechanism of gradual extinction of the activity of the anterior pituitary cells caused by the PROP1 gene mutation may be of importance. Arroyo et al. presented a patient with CPHD-PROP1 with normal height [32]. As a probable cause of this phenomenon, the authors tentatively proposed the presence of GH, although it occurs at low levels in the circulation during childhood and adolescence, the lack of circulating estrogen delaying epiphyseal fusion, resulting in growth beyond the period of normal growth [32]. Despite the absence of a statistically significant difference in mean height at the time of diagnosis in both groups, the CPHD-PROP1 patients presented at a younger age and showed lower growth velocity during the 3 years preceding introduction of growth hormone therapy. Paradoxically, patients in the CPHD-nonPROP1 group were characterized by a better growth rate and thus an older age at the time of diagnosis. This indicates a possible significant role in the growth process of hormonal factors other than the somatotropin axis, most likely of hypothalamic-pituitary origin (the paradox of “growing without growth hormone”) [33–36]. This issue requires further research. The TSH deficit in the CPHD-PROP1 was diagnosed in the younger cases and was more severe than in the CPHD-nonPROP1 group. As some reports have demonstrated, TSH deficiency in CPHD-PROP1 is also highly variable and has been reported as the first presenting symptom in some cases, while others show delayed TSH deficiency [37, 38]. Symptoms of hypogonadism at birth (non-descended testes and/or micropenis) were found less frequently in boys of the CPHD-PROP1 group. Although *PROP1* is essential for the differentiation of gonadotrophs in fetal life, the spectrum of gonadotrophin deficiency ranges from hypogonadism presenting at birth with micropenis and non-descended testes to complete lack of pubertal development or even spontaneous pubertal development with infertility [38–40]. The most interesting phenomenon in patients with the *PROP1* mutation is the occurrence of secondary adrenal insufficiency. It is known that *PROP1* is not directly involved in the transcription of genes necessary for the formation of ACTH. Nevertheless, some authors report late onset of ACTH deficiency in patients with *PROP1* mutations [34, 41]. This phenomenon has been postulated to be a result of dysfunction of PROP1 in initiating pituitary stem cell migration and differentiation [42]. The etiology of this disorder in patients of the CPHD-PROP1 group remains the subject of hitherto unverified hypotheses. A possible mechanism of this phenomenon could be the lack of important paracrine factors normally produced by the cells surrounding the corticotropes and absent in the pituitary of these patients, or progressive corticotrope apoptosis. However, a similar phenomenon was not confirmed in the mouse model of the disorder. Ward et al. showed that *PROP1* in mouse embryos influences the process of migration and metaplasia of the ectodermal to glandular epithelium [43]. Thus, the lack of its influence could adversely affect the morphogenesis and function of the anterior pituitary gland. The pituitary morphology in MRI in CPHD-PROP1 is variable. Most patients have normal pituitary stalk and posterior lobe, while PSIS is typically not observed. The anterior pituitary is normal or enlarged in the early stages of the deficiency and undergoes involution later [44, 45]. The involvement of PROP1 in the abnormal development of the anterior pituitary gland is indirectly supported by the presence of pituitary tumors characterized by an abnormal hypointense signal in T2-dependent MRI scans, which may increase or decrease, leading to hypoplasia of the pituitary gland over time in patients with CPHD-PROP1 [6, 46, 47]. Pituitary enlargement in patients with *PROP1* gene inactivating mutations represents cystic hyperplasia of the intermediate pituitary lobe [48, 49]. In the present study, in the CPHD-PROP1 group, MRI of the pituitary gland revealed decreased pituitary diameter in 21 cases and its enlargement in eight. In nine CPHD-PROP1 patients, the anterior pituitary was normal in MRI, which was also found by Bulut et al. in 6/11 CPHD-PROP1 patients [50]. Surprisingly, in one patient, we found a pituitary stalk interruption and ectopy of the posterior lobe. Regarding this particular group of patients, this observation has not, to our knowledge, previously been described in the literature [11, 51], such an MRI finding being much more common in the CPHD-nonPROP1 group. This observation proves that typical pituitary stalk interruption signs in patients with CPHD do not exclude the diagnosis of CPHD-PROP1 and may indicate the coexistence of CPHD causes other than the *PROP1* mutation in the same patient. It is also worth emphasizing the variability of MRI images and the lack of correlation between pituitary size and pituitary function in CPHD-PROP1 patients [51].

The main limitation of the current study results from its retrospective nature. Because the data were obtained from archival medical records, there are differences in the methodology of biochemical tests and imaging techniques. For the same reason, the oldest patients lack MRI imaging (this was not available at the time of diagnosis). An undoubted limitation of the study is also the lack of some data that were impossible to obtain due to the retrospective nature of the study and long observation period. Another limitation is the fact that the CPHD-nonPROP1 group consists of highly heterogeneous genetic disorders, i.e., mutations of many transcription factors expressed early in pituitary organogenesis resulting in syndromic hypopituitarism and mutations of POU1F1 (Pit1), which, as with Prophet of Pit1 (PROP1), are expressed at a later stage of pituitary organogenesis and result in non-syndromic hypopituitarism.

## Conclusion

Based on our analysis, it can be concluded that in cases of CPHD caused by *PROP1* gene mutations, a normal course of pregnancy and delivery, an absence of dysmorphic features, and normal birth weights are typically observed. The leading symptom occurring in early childhood is a decrease in growth velocity and, consequently, short stature caused by a lack of GH and TSH. A constant feature is hypogonadism, the first symptoms of which (micropenis and cryptorchidism) may be visible in newborn males. Some patients develop ACTH deficiency over time. The morphology of the pituitary can vary, from a reduction in size to enlargement, with no structural abnormalities of the hypothalamic-pituitary region usually observed.

The results show that certain clinical features may allow for the identification of children requiring diagnostics for the PROP1 gene mutation. Despite the retrospective nature of the work, the information contained herein may be clinically useful in the diagnostic process.
