# Correction: Comparison of clinical characteristics of a pediatric cohort with combined pituitary hormone deficiency caused by mutation of the *PROP1* gene or of other origins

**DOI:** 10.1007/s42000-024-00543-0

**Published:** 2024-03-18

**Authors:** Agata Zygmunt-Górska, Małgorzata Wójcik, Aleksandra Gilis-Januszewska, Anna Starmach, Mirosław Bik-Multanowski, Jerzy B. Starzyk

**Affiliations:** 1 Department of Pediatric and Adolescent Endocrinology, University Children’s Hospital in Cracow, Cracow, Poland; 2 Department of Pediatric and Adolescent Endocrinology, Chair of Pediatrics, Pediatric Institute, Jagiellonian University Medical College, Ul. Wielicka 265, Cracow, 30-663 Poland; 3 Chair and Department of Endocrinology, Jagiellonian University Medical College, Cracow, Poland; 4 Department of Medical Genetics, Jagiellonian University Medical College, Cracow, Poland

**Hormones (2023) 23:69-79**

10.1007/s42000-023-00510-1

In this article the author’s name Aleksandra Gilis-Januszewska was incorrectly written as AleksandraGilis-Januszewska.

The original article has been corrected.

